# Suppression of virulence factors of uropathogenic *Escherichia coli* by Trans-resveratrol and design of nanoemulgel

**DOI:** 10.1186/s12866-024-03538-4

**Published:** 2024-10-17

**Authors:** Dalia Saad ElFeky, Abeer Ahmed Kassem, Mona A. Moustafa, Hanan Assiri, Areej M. El-Mahdy

**Affiliations:** 1 Department of Medical Microbiology and Immunology, Faculty of Medicine, Cairo University, Cairo, Egypt; 2 Department of Pharmaceutics and Pharmaceutical Technology, Faculty of Pharmacy, Pharos University in Alexandria, Alexandria, Egypt; 3 Health Sciences Research center, Princess Nourah bint Abdulrahman University, Riyadh, Saudi Arabia; 4 Department of Microbiology and Immunology, Faculty of Pharmacy, Mansoura University, Mansoura, 35516 Egypt

**Keywords:** UPEC, Trans-resveratrol, Virulence factors, Gene expression, Nanoemulgel

## Abstract

**Background:**

Development of multidrug resistance in Uropathogenic *Escherichia coli* (UPEC) makes treatment of Urinary Tract Infections (UTIs) a major challenge. This study was conducted to investigate the effect of trans-resveratrol (t-RSV) at a subinhibitory concentration (sub-MIC-t-RSV) on phenotypic and genotypic expression of virulence factors of clinical isolates of UPEC and develop a nanoformulation of t-RSV. Fifty-five clinical UPEC strains were investigated for the presence of virulence factors by phenotypic methods and PCR detection of virulence genes. The effect of sub-MIC-t-RSV was studied on the phenotypic and genotypic expression of virulence factors. t-RSV-loaded nanoemulgel formulation was prepared and characterized.

**Results:**

Out of the 55 tested isolates, 50.9% were biofilm producers, 23.6% showed both mannose-sensitive and mannose-resistant hemagglutination, 21.8% were serum-resistant, 18.2% were hemolysin producers, while 36.4% showed cytotoxic effect on HEp-2 cells. A total of 25.5% of the isolates harbor one or more of *hly-A*, *cnf-1* and *papC* genes, while 54.5% were positive for one or more of *fimH*, *iss* and *BssS genes*. A concentration of 100 µg/mL of t-RSV effectively downregulates the phenotypic and genotypic expression of the virulence factors in positive isolates. A stable t-RSV-nanaoemulgel with droplet size of 180.3 nm and Zetapotential of -46.9 mV was obtained.

**Conclusion:**

The study proves the effective role of t-RSV as an antivirulence agent against clinical UPEC isolates in vitro and develops a stable t-RSV-nanoemulgel formulation to be assessed in vivo. The promising antibacterial and antivirulence properties of t-RSV place this natural compound to be a better alternative in the treatment of persistent UTIs.

## Introduction

Urinary tract infections (UTI) affects around 150 million people worldwide annually, making it among the most widespread bacterial infections globally [1]. They pose a public health threat in both community and healthcare settings being the most common outpatient infection and additionally, as high as 9.4% of patients in the healthcare settings are affected by healthcare-associated UTIs [2]. Infection may affect the bladder (cystitis) or progress to the kidneys (pyelonephritis). In severe cases, bacteria can reach the bloodstream causing bacteremia [3]. Globally, Uropathogenic *Escherichia coli* (UPEC) is the leading cause of UTIs in both hospital and community settings with significant morbidity and mortality [4].

A variety of virulence factors are encoded by UPEC strains, facilitating their colonization persistence, and pathogenesis in the urinary tract [4], yet no single set of virulence or fitness genes has been identified in all strains of UPEC [3]. Key UPEC virulence factors include fimbriae or adhesins, biofilms, and toxins. In UPEC strains, type I fimbriae or mannose-sensitive hemagglutinins (MSHA) and type P (pyelonephritis-associated) fimbriae or mannose-resistant hemagglutinins (MRHA) are the most common expressed fimbriae [4]. The Binding of type I fimbriae to urothelium mannosylated uroplakin receptors is critical for bladder colonizaton by UPEC. On the other hand, P fimbriae bind to renal epithelial cells glycosphingolipids and are preferentially expressed in UPEC isolates associated with pyelonephritis, [5]. Agglutination of human RBCs is another virulence factor in UPEC infections [6]. UPEC biofilm production promotes bacterial growth as well as persistence at the site of infection by providing an environment rich in nutrients, and protecting bacteria from antimicrobials. Additionally, α-hemolysin, is a pore-forming toxin expressed by UPEC strains associated with more virulence [4]. Hemolysin alters the uroepithelium cytoskeleton causing bladder epithelium shedding and disrupting the function of uroepithelium barrier [5]. Additionally, 30–40% of UPEC isolates express the cytotoxic necrotizing factor type 1 (CNF-1) which is cytotoxic to the uroepithelium and contribute to urothelial invasion [5, 7]. Further, serum resistance is critical for UPEC strains that cause urosepsis [8].

UTIs can only be treated with antibiotics. However, in recent years, multidrug resistance in uropathogens, including UPEC, represents a public health threat especially in patients who suffer recurrent UTIs [9]. It is expected that by 2050, each year, multidrug resistant *E. coli* will cause the death of about 3 million people [10]. This makes it increasingly important to develop new, efficient, and antibiotic-sparing alternatives for UTIs treatment [1]. Recently, a lot of focus has been on finding and choosing naturally occurring bioactive compounds—particularly polyphenols—that work well to both prevent and treat urinary tract infections [11].

In recent years, Trans-resveratrol (t-RSV), a naturally occurring polyphenolic compound, has gained great attention because of its possible health benefits, including antioxidant, anti-aging, and antimicrobial activities. t-RSV has antimicrobial activity against a surprisingly wide range of gram-positive and gram-negative bacteria, in addition to viral and fungal species. Recently, the compound has gained popularity as a nutraceutical as it is well tolerated by humans [12]. It demonstrated antifungal effect against *Candida albicans,* dermatophytes and *Trichosporon beigelii*. Regarding its antibacterial spectrum, RSV at low concentrations, inhibits some bacterial pathogens including *Bacillus cereus*, *Campylobacter coli*, *Helicobacter pylori*, *Neisseria gonorrhoeae* and *Vibrio cholerae*. While at higher concentration, it has inhibitive activity against *Mycobacterium tuberculosis*, *Enterococcus faecalis*, *Streptococcus pyogenes*. Additionally, it has been shown to inhibit Gram-negative bugs including *E. coli*, *Klebsiella pneumoniae, Salmonella* Typhimurium and *Pseudomonas aeruginosa* but at higher concentrations relative to gram positive pathogens [13]. RSV has also antiviral activity against herpes simplex viruses, influenza viruses, respiratory syncytial virus and Ebstein-Barr virus [14].

In most studies that investigate the antimicrobial activity of polyphenols on bacteria, high doses were used that were much higher than their level in a normal diet. As a consequence, these doses while killing pathogens, they also kill gut bacterial flora [15]. Hence, polyphenol concentrations below those toxic to commensal enteric bacteria would be better suited for studying their antibacterial effects [16].

In a study involving two standard strains of UPEC, Lee and his team investigated the antivirulence activities of sub-inhibitory concentrations of t-RSV and some RSV oligomers [6]. According to their findings, t-RSV and oxyresveratrol have the greatest inhibitive properties against UPEC virulence factors and should be further investigated as antivirulence strategies against persistent UPEC infections [11].

The oral administration of t-RSV in treatment of UTIs is challenged by its poor oral bioavailability which is mainly due to its limited water solubility and rapid hepatic metabolism [17]. Nanoemulgel is a novel system prepared by encapsulating a nano-emulsion into a gel system thus improving its stability. The gelling agent can reduce the thermodynamic instability of the emulsion, due to diminished motion of the non-aqueous phase because of the elevated consistency of the external medium. Nanoemulgel enhances drug delivery for both immediate and controlled release, and conveys the advantages of both hydrogels and nano-emulsions [18, 19]. Moreover, choosing a mucoadheive hydrogel can ensure better retention inside the urinary bladder. Nanoemulgel is a suitable carrier system for delivery of t-RSV as it has a good solubilizing effect on lipophilic drugs and hence, can cause significant improvement in drug loading capacity [20].

In view of this, the current research was conducted to study the antivirulence therapeutic potential of t-RSV in persistent UTI through assessing the inhibitory effect of this compound at subinhibitory concentration on phenotypic and genotypic expression of virulence factors of clinical isolates of UPEC. Additionally, to overcome the t-RSV’s poor oral bioavailability, our work aimed to develop and characterize an t-RSV nanoemulgel for direct intrauretheral application. To the best of our knowledge, this is the first investigation to assess t- RSV as anti-virulence agent on a large number of clinical UPEC isolates.

## Materials and methods

**Fig. 1:**
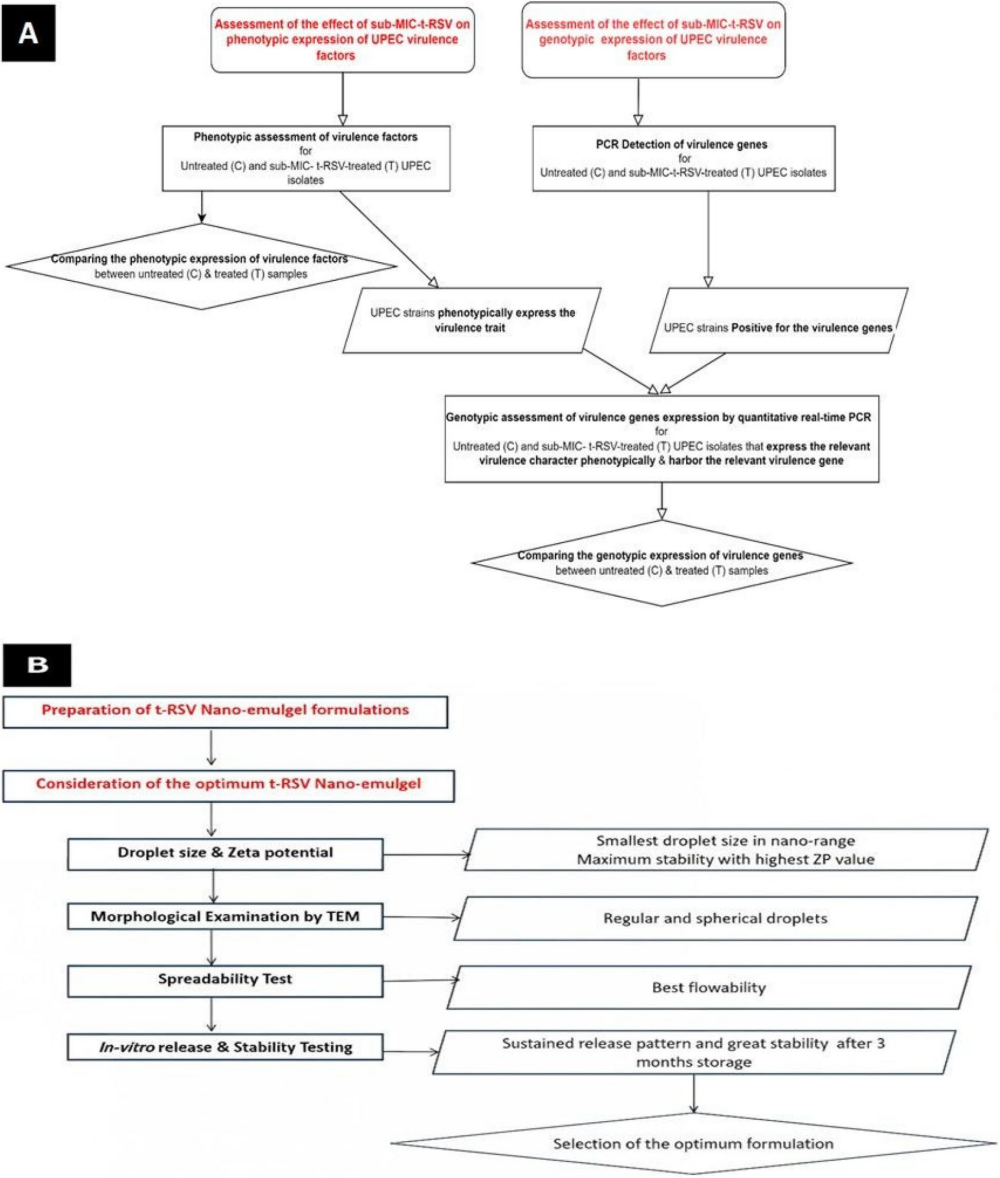
Flow chart for the study protocol.; **A**: Effect of Sub-MIC-t-RSV on phenotypic and genotypic expression of UPEC clinical isolates; **B**; Development of t-RSV nanoemulgel

Bacterial isolates included in the study were provided by the Microbiology Lab at King Abdullah bin Abdulaziz University Hospital (KAAUH), Princess Nourah bint Abdulrahman University (PNU), Riyadh, Saudi Arabia, from January to June 2021. The practical part of the study was conducted in the Department of Medical Microbiology and Immunology, Faculty of Medicine, Cairo University, Egypt; The Department of Microbiology and Immunology, Faculty of Pharmacy, Mansoura University, Egypt; The Pharmaceutical Nanotechnology Research Lab (PNRL), Faculty of Pharmacy, Pharos University, Alexandria, Egypt, while the cytotoxicity assay experiment was conducted in the Health Sciences Research Center (HSRC), PNU, Riadh, Saudi Arabia. Schematic presentation of the study protocol is shown in Fig. 1.

### Trans-t-RSV

t-RSV powder (98% pure) was obtained from Hi-tech Development Zone, XI’AN, China. Stock solution was prepared by adding 0.06 g of t-RSV to 3 mL of dimethyl sulfoxide (DMSO). Stock solution was stored at 4 °C for up to 48 h. Working solution (12 mg/mL) was prepared just before use. The chosen concentration of DMSO in the working solution (0.5%) has shown no bactericidal activity by broth microdilution method (BMD).

### Bacterial isolates

A total of 55 clinical UPEC strains isolated from urine cultures of inpatients and outpatients presented with UTIs at KAAUH were included in the study. Identification of the isolates was done using Vitek II system. Isolates were inoculated in brain heart infusion (BHI) broth supplemented with 10% glycerol and stored at -80 °C until further testing.

### t-RSV minimum inhibitory concentration assay and the effect of sub-inhibitory concentrations on bacterial count

The minimum inhibitory concentration (MIC) of t-RSV against UPEC isolates was determined using the standard BMD described by the Clinical Laboratory Standards Institute (CLSI) [21]. In brief, bacterial suspensions from each of the included isolates were prepared from fresh overnight cultures and adjusted to 0.5 MacFarland. Sock solution of t-RSV was diluted in BHI to get a final concentration of 12 mg/mL as described above. In a 96-well round bottom plate, a serial twofold dilutions of t-RSV (2000 – 12.5 μg/mL) were prepared and 10 µL from the prepared bacterial suspensions were added to each well. Plain BHI with and without t-RSV were used as a control. The MIC was determined as least concentration with no visible bacteria growth in the broth. The viability of tested isolates was evaluated by tenfold serial dilution of t-RSV in LB broth at two subinhibitory concentrations of t-RSV (100 and 200 µg/mL) to determine the working antivirulence concentration of the compound [22]**.**The OD of the surviving UPEC isolates with both concentrations of t-RSV was compared to the untreated cells cultivated under the same conditions. This was also confirmed by viable count using the pour plate method.

### Effect of sub-MIC of t-RSV on phenotypic expression of virulence factors of UPEC isolates

In the current study, the antivirulence activity of sub-MIC**-** t-RSV was assessed against some of the most important UPEC virulence factors including adhesins, biofilm formation, serum resistance, cytotoxicity and hemolysin production. Before each of the following experiments; stored bacterial isolates were subcultured on BHI agar at 37 °C for 18–24 h. For each isolate, 2 bacterial suspensions were prepared and adjusted to 0.5 MacFarland in 2 tubes; one of them were used as the control (C) and the other tube (T) was treated with the chosen sub-MIC of t-RSV. Both the C and T tubes were incubated overnight on a shaker incubator at 37° C and 150 rpm to be used for the following experiments. For the hemagglutination experiment, the incubation extended to 48 h for full fimbriation.

#### Biofilm formation

Quantitative biofilm assay was performed using microtiter plate assay in triplicate for both control (C) and t-RSV-treated (T) cultures as previously described [23, 24]. The optical density (OD) was measured at 492nm using Bio-RAD, xMark microplate spectrophotometer. For each isolate, the mean OD of both control (C) and treated (T) of the 3 wells were calculated (OD_T_). The mean OD of the negative control was also calculated. *E. coli* ATCC 25922 was used as a positive control for biofilm formation.

#### Haemagglutination

Hemagglutination was detected by the ability of both control (C) and treated (T) bacterial suspensions to agglutinate group “O” RBCs in presence and absence of D-mannose by direct bacterial hemagglutination test–slide method as previously described [25]. RBCs suspension with and without D-mannose were used as negative control, while *E. coli* ATCC 25922 was used as a positive control for MRHA. Presence of clumping indicates hemagglutination.

#### Serum resistance

Serum resistance for both control and treated isolates was tested as previously described [26]. For each isolate, both control (C) and treated (T) overnight bacterial suspensions were adjusted to 2.5 × 10^4^ CFU/mL. On BHI agar plates, 10µL of the suspension were inoculated (zero time, T0). Next, 50 µL of bacterial suspensions were mixed with equal quantities of human serum, incubated at 37°C for 3 h (T3), and then 10 µL were inoculated on BHI agar (T3). Both plates (T0 and T3) were incubated overnight at 37°​C to determine viable bacterial count. A serum-resistant strain was defined as one in which greater than 90% of organisms survived after 3 h, while a serum-sensitive strain was defined as one that dropped to 1% of the initial count. *E. coli* ATCC 25922 was used as a positive control for serum resistant phenotype.

#### Hemolysin production

Screening for hemolysin production was performed qualitatively as previously described [27]. Bacterial suspensions from overnight fresh cultures adjusted to 0.5 McFarland were inoculated on 5% sheep blood agar and incubated overnight at 37°C. Isolates showing complete hemolysis on blood agar were further evaluated quantitatively for hemolysin production as previously described [28]. Briefly, overnight broth culture with (T) and without t-RSV (C) in BHI were incubated on a shaker incubator at 37 °C and 150 rpm. Cell free extracts were obtained by centrifugation at 10,000 rpm for 10 min at 4°C. Aliquot of 600 µL of fresh RBCs suspension (2% RBCs in PBS) was mixed with equal quantities of cell free extract and incubated at 37 °C for 2 h. with mild agitation. Centrifugation at 10,000 rpm for 10 min at 4°C was done and hemoglobin release was assessed by measuring OD at 540 nm. Negative control (RBCs suspension without supernatant) and positive control (0.2% Sodium dodecyl sulphate) were included in the test. Hemoglobin release was calculated from the following equation:

$$\%\;\mathrm{of}\;\mathrm{Hemoglobin}\;\mathrm{release}\;=\frac{\mathrm{Absorbance}\;\mathrm{of}\;\mathrm{sample}\;-\;\mathrm{Absorbance}\;\mathrm{of}-\mathrm{ve}\;\mathrm{control}\;}{\mathrm{Absorbance}\;\mathrm{of}\;+\;\mathrm{ve}\;\mathrm{control}\;-\;\mathrm{Absorbance}\;\mathrm{of}\;-\;\mathrm{ve}\;\mathrm{control}}\times100$$

#### Cytotoxic activity

Bacterial colonies from overnight cultures on BHI agar were inoculated in 10 mL of BHI broth of both treated (T) and control (C) and incubated overnight on shaker incubator at 37ºC and 150 rpm. Bacteria were harvested by centrifugation at 10.000 rpm at 4°C to be used for qualitative detection of cytotoxin production on HEp-2 cells; a human laryngeal squamous carcinoma cell line (ATCC-CCL 23); purchased from ATCC supplied by Vacsera, Egypt [29, 30]. The supernatant was filtered using sterile bacterial membrane syringe filter pore size 0.22 µm (Biomed Solutions) and the filtrate was used for quantitative detection using CyQUANT™ MTT Cell Proliferation Assay Kit (Invitrogen, Thermo Fisher Scientific, USA). DMEM (Dulbecco's Modification of Eagles Medium) UFC Biotech, KSA, supplemented with 2% fetal bovine serum (FBS), 100U/mL penicillin, and 100g/mL streptomycin was used to maintain HEp-2 cells at 37°C in 5% CO_2_with subculture every 3–4 days. A confluent monolayer of HEp-2 cells was formed in both 24-well and 96- well tissue culture plates after 24 h of seeding the cells suspended in DMEM with 10% FBS at 37°C with 5% CO_2_.

For qualitative assessment of the effect of the UPEC isolates on HEp-2 cells, bacterial pellets of each of the (C) and (T) isolates were washed with PBS, resuspended in DMEM, and adjusted to a count of 1 × 10^8^ CFU/ mL. Aliquots of 100 µl of the prepared bacterial cell suspensions were added to each well of a 24-well plate seeded with HEp-2 cells and incubated at 37ºC in 5% CO_2_ for 3 h. Each of the (C) and (T) suspensions of each isolate were tested in duplicate. HEp-2 cell morphology was assessed 1-, 2- and 3-h after infection by an inverted microscope (Optech, Germany). Images were captured using a camera (Canon powershot A650) attached to the inverted microscope (Mag × 400). Negative control (HEp-2 cells without bacteria) was included.

MTT assay was performed according to manufacturer’s instructions. Each of the (C) and (T) suspensions of each isolate were tested in duplicate. Briefly, 100 µL of bacterial filtrate were added to each well of a 96- well microplate seeded with HEp-2 cells and incubated at 37°C in 5% CO_2_. After 2 h, the contents of the wells were aspirated and discarded and 100 µL of fresh media and 10 µL of MTT (5 mg/mL) were added and incubated at 37°C in 5% CO_2_. After 4 h, the content of the wells was aspirated and discarded and DMSO (100 µL) was added to each well. After shaking for 10 min at 100 rpm/min, OD were measured at 540 nm. Cytotoxicity percentage of both (C) and (T) cultures was calculated from the following formula:

$$\%\;\mathrm{of}\;\mathrm{Cytotoxicity}=\left[100\times\left(\mathrm{Control}-\mathrm{Sample}\right)\right]$$

### PCR detection of UPEC virulence genes

Thermal extraction of DNA from the isolates was performed as previously described [31]. Two multiplex PCR reactions were used to detect *E. coli* virulence genes using primers listed in (Table 1A) [32, 33]. The first reaction detects mannose-sensitive haemagglutination “MSHA” (*fimH*), serum resistance (*iss*) and biofilm formation (*BssS*) genes, while the second one detects hemolysin (*hly-A*), cytotoxicity(*cnf-1*), and mannose-resistant haemagglutination “MRHA” (*papC*) genes. For both reactions, in a total volume of 20 µL, the followings were added, 4 µl of HOT FIREPol® MultiPlex Mix with 10 mM MgCl2, 5x (Solis BioDyne, Estonia), 1 µl of both forward and reverse primer for each gene (400nM), 1 µl of the DNA template and 9 µl of nuclease free water. Negative control (nuclease free water instead of template DNA) was included in each run. During the first reaction, denaturation was carried out at 95°C for 12 min followed by 40 amplification cycles (denaturation at 94°C for 40 s., annealing at 50°C for 1 min., and extension at 72°C for 1 min.) and a final extension at 72°C for 5 min.For the second reaction, we performed a denaturation step at 95°C for 12 min, followed by 30 cycles of amplification (denaturation at 94°C for 1 min, annealing at 56°C for 1 min, and extension at 72°C for 1 min), followed by a final extension at 72°C for 5 min.

**Table 1:** Primer used for the tested virulence genes of *E. coli*

A. Multiplex PCR				
**Gene name**	**Sequence (5′ → 3′)**	**Annealing Temperature**	**Product size** **Bp**	**Reference**
***fimH***	F: TACTGCTGATGGGCTGGTC	50°C	640	[32]
	R: GCCGGAGAGGTAATACCCC			
***Iss***^*a*^	F: GGCAATGCTTATTACAGGATGTGC		260	
	R: GAGCAATATACCCGGGCTTCC			
***BssS***^*a*^	F: GATTCAATTTTGGCGATTCCTGC		225	
	R: TAATGAAGTCATTCAGACTCATCC			
***hly-A***	F: AACAAGGATAAGCACTGTTCTGGCT	56°C	1177	[33]
	R: ACCATATAAGCGGTCATTCCCGTCA			
***cnf-1***	F: AAGATGGAGTTTCCTATGCAGGAG		498	
	R: CATTCAGAGTCCTGCCCTCATTAT			
***PapC***^*a*^	F: GACGGCTGTACTGCAGGGTGTGGCG		328	
	R: ATATCCTTTCTGCAGGGATGCAATA			
**A. Quantitative Real-time PCR (qPCR)**		**Reference**		
*V1 to V6 region of 16S-rRNA*	F: AGAGTTTGATCMTGGCTCAG	[32]		
	R: ACGAGCTGACGACARCCATG			
*fimH*	F: TACTGCTGATGGGCTGGTC			
	R: TCGTTATGGCAAAAGATTTGCGT			
*hly-A*	F: AACAAGGATAAGCACTGTTCTGGCT	This study		
	R: ACCATATAAGCGGTCATTCCCGTCA			
*cnf-1*	F: AAGATGGAGTTTCCTATGCAGGAG			
	R: CATTCAGAGTCCTGCCCTCATTAT			

*F* Forward, *R* Reverse

^a^The same primers were used for real-time PCR

### Effect of sub-MIC of trans-t-RSV on the expression of UPEC virulence genes

The effect of t-RSV on the relative expression of virulence genes was assessed for selected untreated (C) and t-RSV-treated (T) UPEC isolates that harbor the relevant gene by PCR and were positive phenotypically for the relevant virulence character. RNA extraction was done by RNeasy mini kit (Qiagen) following the manufacturer’s instructions. RT- PCR was done by High-Capacity cDNA Reverse Transcription Kits, Applied Biosystems. Quantified expression of virulence genes was measured by quantitative Real -Time PCR using HOT FIREPol SolisGreen qPCR mix, 5 × following manufacturer’s instructions using home designed primers listed in Table 1B. The reaction was conducted using Quantstudio 5 (Applied biosystems, Thermo Fischer Scientific). The formula R = 2^−ΔΔCt^ was used to calculate the normalized value of the level of expression in comparison to the calibrator [34]. ΔΔCt = sample ΔCt—average control group ΔCt, while ΔCt = target gene Ct—housekeeping gene Ct.

### Preparation of t-RSV formulations

#### Preliminary screening for nanoemulgel preparation

Different surfactants (Tween 20, Tween 80, Span 20, and Span 80) were added to 50 mL of oily phase (liquid paraffin). Each mixture was quietly heated at 50°C for complete homogenization of the ingredients. Simply, 1 mL of each oil-surfactant combination was diluted to 25 mL using deionized water. The revealed mixture was visually evaluated for turbidity and transmittance using UV spectrophotometer (Shimadzu-1700, Japan)at λ max 638 using filtered distilled water as blank [35].

#### Preparation of t-RSV-loaded nanoemulgel

Carbopol 974 (obtained from BF Goodrich Company, USA) uniform gel (0.5% w/v) in water was obtained by continuous stirring using magnetic stirrer (JeioTech TM-14SB, UK). An accurately weighed amount of t-RSV (0.1% w/v) was dissolved in analytical grade liquid paraffin to form the oily phase. Propylene glycol (PG) as a co-surfactant was added to 20 mL water and then vortexed for 300 s. After that, the aqueous phase was added to the oily phase under continuous stirring till forming the primary emulsion. t-RSV-loaded emulsion was homogenized at 10,000 rpm for 5 min after which carbopol gel was added and mixed [19]. To ensure the gelling of carbopol 974, the pH of the solution was adjusted by the addition of triethanolamine (TEA) drop by drop to pH 7 and then the formulations were stored at 4 Cº for further investigation. Composition of t-RSV-loaded nanoemulgel formulations is illustrated in Table 2.

**Table 2:** Composition, droplet size analysis and zeta potential of t-RSV formulations

Formula code	t-RSV (%w/v)	Carbopol 974 (%w/v)	Liquid paraffin (mL)	Tween 80 (mL)	Tween 20 (mL)	Propylene glycol (mL)	Water to (mL)	Droplet size (nm) ± SD	Zeta potential (mV) ± SD	PDI ± SD
F1	0.1	0.5	5	1	-	1	50	180.3 ± 1.22	-46.9 ± 0.1	0.31 ± 0.015
F2	0.1	0.5	5	-	1	1	50	263.5 ± 3.12	-40.4 ± 0.11	0.48 ± 0.012
F3	0.1	0.5	5	0.5	0.5	1	50	220 ± 2.82	-42.3 ± 0.05	0.39 ± 0.013

#### Characterization of RSV-loaded nanoemulgel formulations

##### ***Physical assessment***

The fabricated nanoemulgels were examined physically for their color, consistency, and homogeneity.

##### Droplet size analysis and zeta potential studies

The droplet size (DS) and zeta potential (ZP) of the organized nanoemulgel (F1, F2 and F3) were measured using Malvern’s zetasizer. Samples were diluted with filtered distilled water then sonicated for 10 min directly before analysis. Results were detained in triplicates as mean value ± SD.

##### Rheological studies and spreadability test

The viscosity of the prepared formulations was tested at room temperature by means of Brookfield viscometer (RV DV-II + Pro, Brookfield Engineering Labs, USA) [36]. A sample of the nanoemulgel was sheared at different velocities, using spindle 15. Measurement was taken over speeds of 10, 15, 22, 50 and 100 rpm for 1 min. Results were manipulated in triplicates as mean value ± SD. Spreadability was examined by calculating the distribution diameter of 0.3 g of each nanoemulgel between two parallel glass slides for about 3 min till no more spreading observed [35].

##### Morphological study using transmission *electron* microscope (TEM)

Morphological inspection was conceded to the optimized t-RSV-loaded nanoemulgel (F1) by transmission electron microscope (TEM). Fabricated samples were diluted and fixed onto a carbon-coated copper grid. Later, the grid was left for 60 s to let the formulation adherence on the carbon substrate. Staining using 2% uranyl acetate in ethanol (w/v) was applied and dried samples were instantly transferred for TEM examination [35].

##### In-vitro release and kinetics analysis

Drug release from the t-RSV-solution, t-RSV-gel and t-RSV-loaded nanoemulgel (F1) was studied using dialysis method [37]. Dialysis bags were tied from both ends, filled with 500 μL sample equivalent to 500μg of t-RSV and then immersed in 10 mL of phosphate buffer pH 7.4 in addition to 10% (v/v) ethanol to maintain the sink condition [38].The test was performed via controlled shaking water bath at 37 ºC ± 0.5 and 100 rpm. At identified time intervals (0.5, 1, 2, 3, 4, 6 and 24 h.); a sample was taken and the same volume of fresh medium was added instead. After that, samples were measured spectrophotometrically at 306 nm using the release medium as a blank. Experiments were examined in triplicate and outcomes fitted as mean value ± SD. The in-vitro release records were investigated by DD solver software and fitted to kinetic models including; zero order, first order, Higuchi and Peppas—Korsmeyer to express the release kinetics.

##### ***Stability study***

Phase departure, cracking or creaming of the nanoemulgels were distinguished by using centrifugation test [35].The experiment was checked at 10,000 rpm using cooling centrifuge (Centurion Scientific Ltd, UK) at 0°C for 5 min. Then, the selected formulation (F1) was stored in a tightly closed glass vial and kept at 4 ºC over a period of 90 days. The physical features, droplet size, and zeta potential were examined.

### Statistical analysis

A statistical package for the Social Sciences (SPSS) version 28 (IBM Corp., Armonk, NY, USA) was used for coding and entering the data. Quantitative data was summarized by mean, standard deviation, median, minimum, and maximum, while qualitative data was summarized by frequency (count) and relative frequency (percentage). Instead, the exact test was used when the anticipated frequency was < 5 [39].

## Results

The goal of the current investigation was to examine the impact of t-RSV on phenotypic and genotypic expression of virulence factors of 55 clinical UPEC strains isolated from urine samples of 49 cases of cystitis (89.1%) and 6 cases of pyelonephritis (10.9%). Majority of the patients were females (*n* = 50; 90.9%). The age of the patients ranged from 1 to 73 years old with mean of 29.68 ± 20.42.

### t-RSV minimum inhibitory concentration assay and the effect of sub-inhibitory concentrations on bacterial count

The MIC was 500 µg/mL for all the tested isolates. Two subinhibitory concentrations of t-RSV (100 and 200 µg/mL) were assessed for their effect on the bacterial viability. A sub-inhibitory concentration of 100 µg/mL of t-RSV (sub-MIC-t-RSV_100_) was selected to be used in the assessment of the effect of t-RSV on virulence factors of the tested isolates as it was shown that isolates had nearly the same bacterial count of (150 × 10^6^ CFU/mL) compared to that of the control cultures (156 × 10^6^ CFU/mL).

### Effect of t-RSV on phenotypic expression of virulence factors of UPEC isolates

#### Biofilm formation

The development of biofilm was noted in 28 (50.9%) of the examined isolates. Sub-MIC-t-RSV_100_- decreased biofilm formation in all positive isolates by 9 – 69% as shown in Table 3A.

**Table 3:** Biofilm production (A) and %Hemoglobin release (B) of the control and t-RSV treated positive isolates & the percent reduction after t-RSV treatment

	(A) Biofilm production		
Isolate No	Biofilm production		
	Control	t-RSV	% Reduction
2	0.1	0.07	31
3	0.11	0.06	39
6	0.11	0.073	37
7	0.13	0.06	46
10	0.16	0.07	51
14	0.14	0.05	60
16	0.18	0.11	34
18	0.13	0.06	49
19	0.17	0.05	66
21	0.13	0.11	9
22	0.18	0.06	64
23	0.13	0.06	45
26	0.17	0.15	12
28	0.18	0.06	66
30	0.2	0.12	38
31	0.11	0.05	49
35	0.24	0.18	23
40	0.13	0.06	52
42	0.11	0.09	19
43	0.14	0.06	55
50	0.14	0.09	34
51	0.18	0.08	52
61	0.18	0.06	62
63	0.19	0.07	59
69	0.27	0.12	56
70	0.25	0.15	37
86	0.3	0.09	69
96	0.14	0.11	23
	(B) %Hemoglobin release		
Isolate No	% Hemoglobin Release		
	Control	t-RSV	% Reduction
6	35.76	1.1	96.8
13	73.8	59.2	19.8
35	97.8	80	20
40	73.53	23	68.6
50	35.61	29.3	17.7
51	86.83	49.1	43.5
64	98.8	59.76	48
75	36.2	5.3	85
69	96.8	84	13.2
73	40.1	18	55.3

#### Hemagglutination

Out of the 55 tested *E. coli* isolates, 13 (23.6%) isolates showed hemagglutination (HA) of group O RBCs, while 42 (76.4%) were non-hemagglutinating. All positive isolates showed both MRHA & MSHA. It was observed that sub-MIC-t-RSV_100_ markedly inhibited RBCs agglutination in all positive isolates.

#### Serum resistance

According to Vaish et al. [26], results revealed that 12 out of 55 isolates (21.8%) are serum-resistant, where greater than 90% of organisms survived after 3 h. Sub-MIC-t-RSV_100_ had markedly decreased the serum resistance of these isolates by 50–95%.

#### Hemolysin production

Qualitative assessment of hemolysin production showed that 10 (18.2%) of investigated isolates were positive hemolysin producers. Table 3B revealed that sub-MIC-t-RSV_100_ decreased hemolysin production in all positive isolates with varying degree from 13.2 – 96.8% as detected by the quantitative method.

#### Assessment of cytotoxic activity

##### Qualitative assessment of cytotoxic activity

**Fig. 2:**
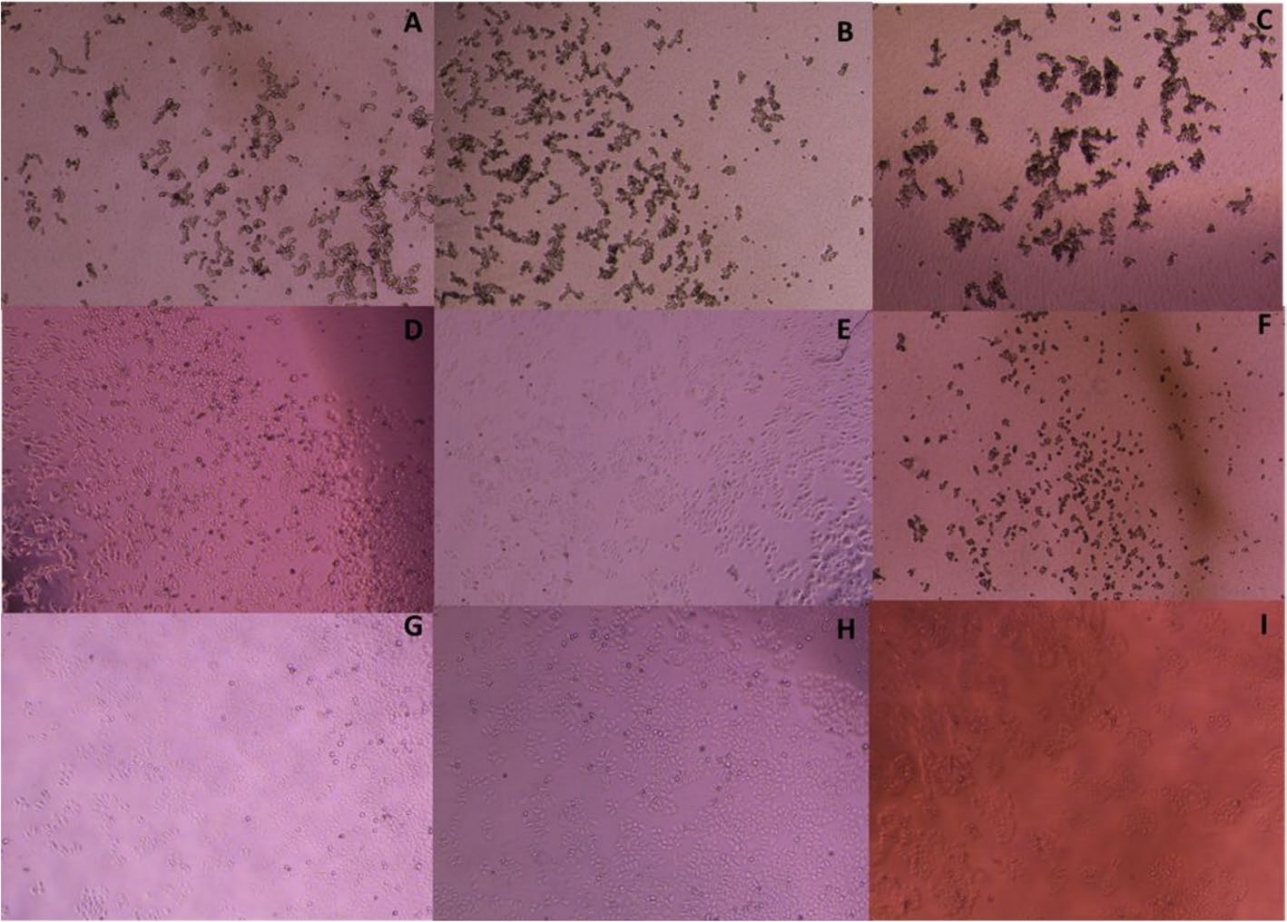
Cytotoxic effect of treated and untreated UPEC isolates on HEp-2 cell line. **A-C**: The cytotoxic effect of the *E. coli* isolate No. 6 on HEp-2 cells after 1h (**A**), 2h (**B**), and 3h (**C**) with complete detachment after 3h of infection; **D-F**: The cytotoxic effect of the *E. coli* isolate No. 6 treated with t-RSV onHEp-2 cells after 1h (**D**), 2h (**E**), and 3h (F) showing weak detachment after 3h of infection. **G-I**: HEp-2 cell line, control cell line after 1h (**G**), 2h (**H**), and 3h (**I**)

Microscopic evaluation of the HEp-2 cell line infected with 1 × 10^8^ CFU/mL revealed that 20 (36.4%) out of 55 UPEC isolates showed cytotoxic effect with varying degree. In all the positive samples, rounding of the HEp-2 cells was observed as early as 1 h after infection, while complete detachment of the cells was observed 3 h after infection as shown in Fig. (2 A-C). On the other hand, 35 (63.6%) isolates did not change the cell line morphology. Additionally, t-RSV had markedly lessened the cytotoxic effect on HEp-2 cell line in the treated isolates (T) that have cytotoxic effect as shown in Fig. (2 D-F) when compared to the same untreated isolates (C). It was observed that the uninfected control HEp-2 cell line showed preserved morphology throughout the experiment (Figs. 2 G-I).

##### ***MTT cytotoxic assay***

Quantitative assessment of the effect of t-RSV on cytotoxicity of UPEC isolates was performed by MTT assay. Results revealed that the 20 isolates that were positive by the qualitative cytotoxic assay have cytotoxic effect on HEp-2 cell after 2 h of infection ranging from (12–35%). On the other hand, the rest of the isolates (*n* = 35) showed no effect on the cell line. Upon treatment with t-RSV, cytotoxicity was decreased in the positive isolates by (9- 77%) as shown in Table 4.

**Table 4:** Cytotoxicity of the positive isolates after 2 h HEp-2 cell infection of Control and t-RSV treated isolates & the percent cytotoxicity decrease after t-RSV treatment

Isolate No	% Cytotoxicity	% Cytotoxicity after t-RSV treatment	% Cytotoxicity reduction by t-RSV
1	18.05	9.71	46
3	11.54	3.8	67
4	24.48	19.14	22
5	23.3	19.38	17
6	22.46	18.98	15
7	23.82	20.96	12
8	17.63	4.15	76
10	29.23	22.13	24
11	26.12	14.05	46
12	35.11	25.33	28
13	33.56	30.41	9
35	13.03	7.14	45
40	31.51	26.64	15
50	32.34	25.23	22
51	24.39	12.48	49
64	32.36	27.78	14
69	28.41	18.7	34
73	27.08	22.88	16
75	28.61	25.43	11
96	19.61	9.37	52

### PCR detection of UPEC virulence genes

**Fig. 3:**
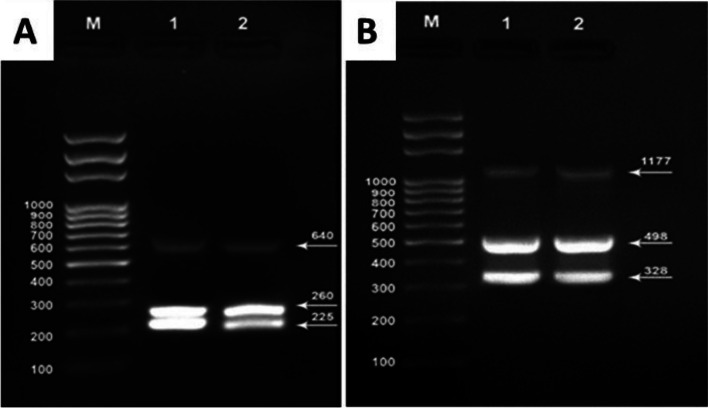
Multiplex PCRs for detection of virulence genes. **A**: Multiplex PCR-1: Lane M: DNA ladder; Lanes 1 & 2: samples positive for *BssS* gene (225 bp), *iss* gene (260 bp) and *fimH* gene (640 bp). **B**: Multiplex PCR-2: Lane M: DNA ladder; Lanes 1 & 2: samples positive for *PapC* gene (328 bp), *cnf-1* gene (498 bp) and *hly-A* gene (1177 bp)

Virulence genes of tested UPEC isolates were detected using 2 multiplex PCR reactions. The first reaction detects *fimH*, *iss* and *BssS* genes, while the second one detects *hly-A*, *cnf-1*and *papC* genes (Fig. 3).

For the first multiplex PCR reaction, in total 30 isolates (54.5%) harbor one or more of these genes;13 isolates were positive for the three virulence genes (*fimH*, *iss* and *BssS*), 8 isolates were positive for both *fimH* and *BssS* genes, 8 isolates were positive for both *iss* and *BssS* genes and only 1 isolate showed the presence of *fimH* gene alone (Fig. 3A). It was found that only 5 out 13 MSHA positive isolates harbor *FimH* gene. In addition, 9 out of the 12 serum resistant isolates harbor *iss* gene and 16 out of the 28 biofilm producing isolates harbor *BssS* gene.

The second multiplex PCR reaction showed that in total 14 isolates (25.5%) harbor one or more of these genes (Fig. 3B); 11 isolates were positive for the three virulence genes (*hly-A*, *cnf-1* and *papC*), 2 isolates showed the presence of both *papC* and *hly-A,* while 1 isolate had only *papC.* All the 10 hemolysin producing isolates detected phenotypically were harboring *hly-A*gene. On the other hand, all the 11 isolates that harbor *cnf-1* gene showed cytotoxicity on HEp-2 cells, however, 7 isolates showed cytotoxicity did not harbor the gene. Only 8 of the 13 MRHA positive isolates harbor the *papC* gene. In addition, 8 out of the 28 biofilm producing isolates harbor *papC* gene, 7 of them contain *BssS* gene as well.

There is a statistically significant association between the presence of both *papC* and *cnf-1* and the presence of *hly-A* and *iss* genes (*p* value ≤ 0.05). In addition, there is a statistically significant association between the presence of *BssS* gene and both *fimH* and *iss* genes (*p*-value ≤ 0.05).

Statistical analysis for the association between the clinical presentation (cystitis/pyelonephritis) and the presence of these virulence gens showed that pyelonephritis is statistically associated with *hly-A*, *cnf-1* and *papC*genes (*p*-value = 002, 0.01 and 0.03, respectively).

### Effect of t-RSV on the gene expression of UPEC

**Fig. 4:**
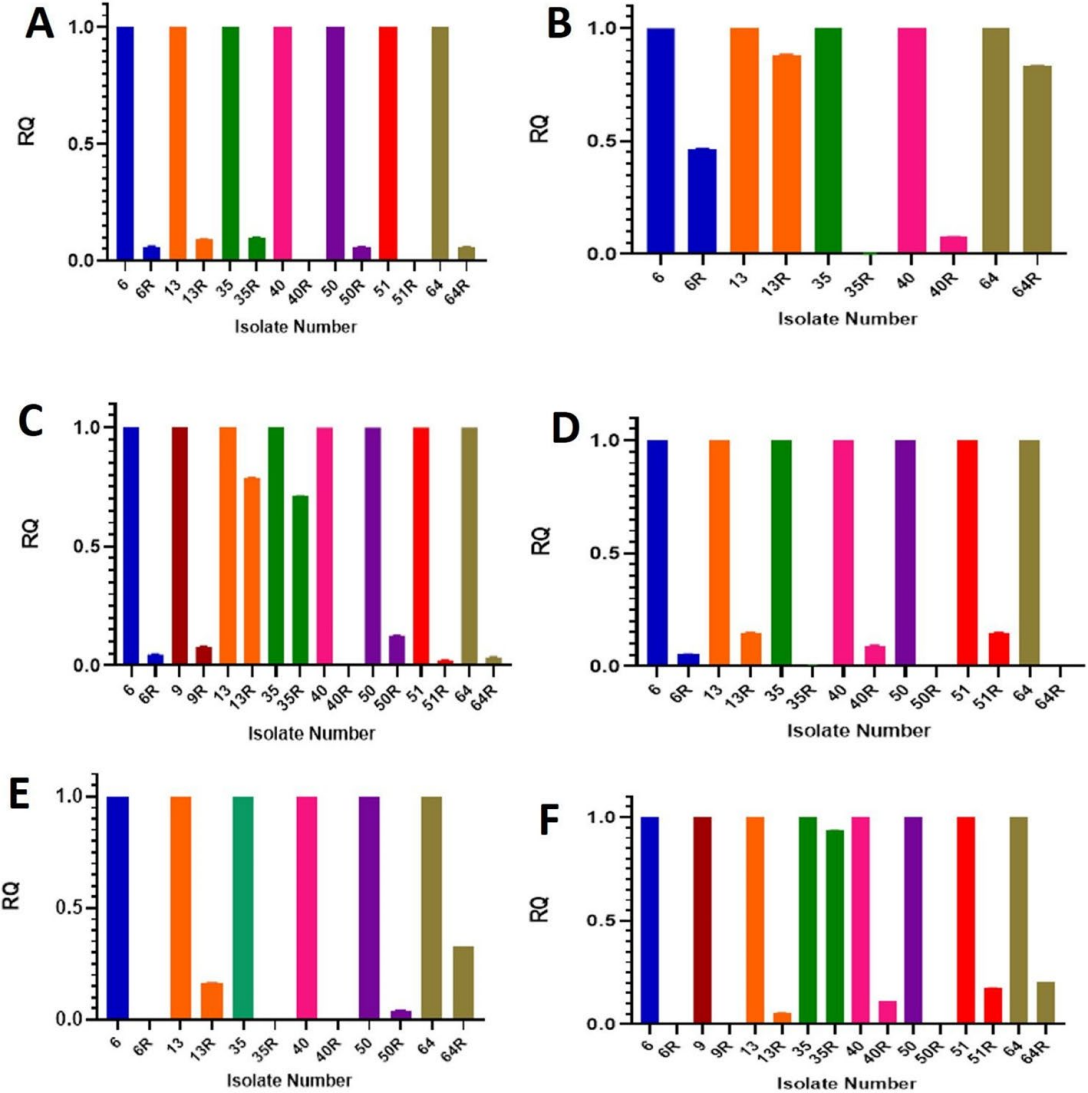
Effect of t-RSV on Relative Expression of Virulence Genes. **A**: Effect on *BssS* gene. **B**: Effect on *fimH* gene (**C**): Effect on *papC* gene. **D**: Effect on *iss* gene. **E**: Effect on *hly-A* gene (**F**): Effect on *cnf-1* gene. In all figures, the mean values and standard deviation of the level of expression of the relevant gene of the triplicate of each treated samples were compared to the control (untreated samples). “RQ = relative quantification”

The effect of t-RSV on the expression of the studied virulence genes was assessed by measuring the relative gene expression of the tested genes in selected untreated (C) and t-RSV-treated (T) UPEC isolates. The selected isolates were shown to harbor the relevant gene by PCR and phenotypically express the relevant virulence character. It was found that sub-MIC-t-RSV_100_has significantly reduced the expression of all the studied genes in the selected positive isolates (Table 5 &Fig. 4).

**Table 5:** Effect of t-RSV on relative expression of virulence genes

Sample no	% decrease of Relative Gene Expression					
	***BssS***	***fimH***	***papC***	***iss***	***hly-A***	***cnf-1***
6	94	53.4	95.4	94.7	100	99.8
9			92			100
13	90.9	11.9	21.2	85.1	83.4	94.4
35	90.2	99.8	28.7	99.7	99.7	6.3
40	99.5	92.5	99.2	90.8	99.6	88.7
50	94.2		87.4	99.3	95.7	99.4
51	99.97		97.9	85.1		82.4
64	94.3	16.8	96.6	99.5	67.3	79.3

### Results for the nanoemulgel formulation

#### Physical assessment

All the fabricated nanoemulgels were white in color, viscous, washable, smooth formulations with homogeneous consistency. But for centrifugation test, only three formulae; F1, F2 and F3 showed good physical stability with no phase separation, creaming or cracking, so that they were transferred for further optimization.

#### Droplet size analysis and zeta potential and surface morphology

**Fig. 5:**
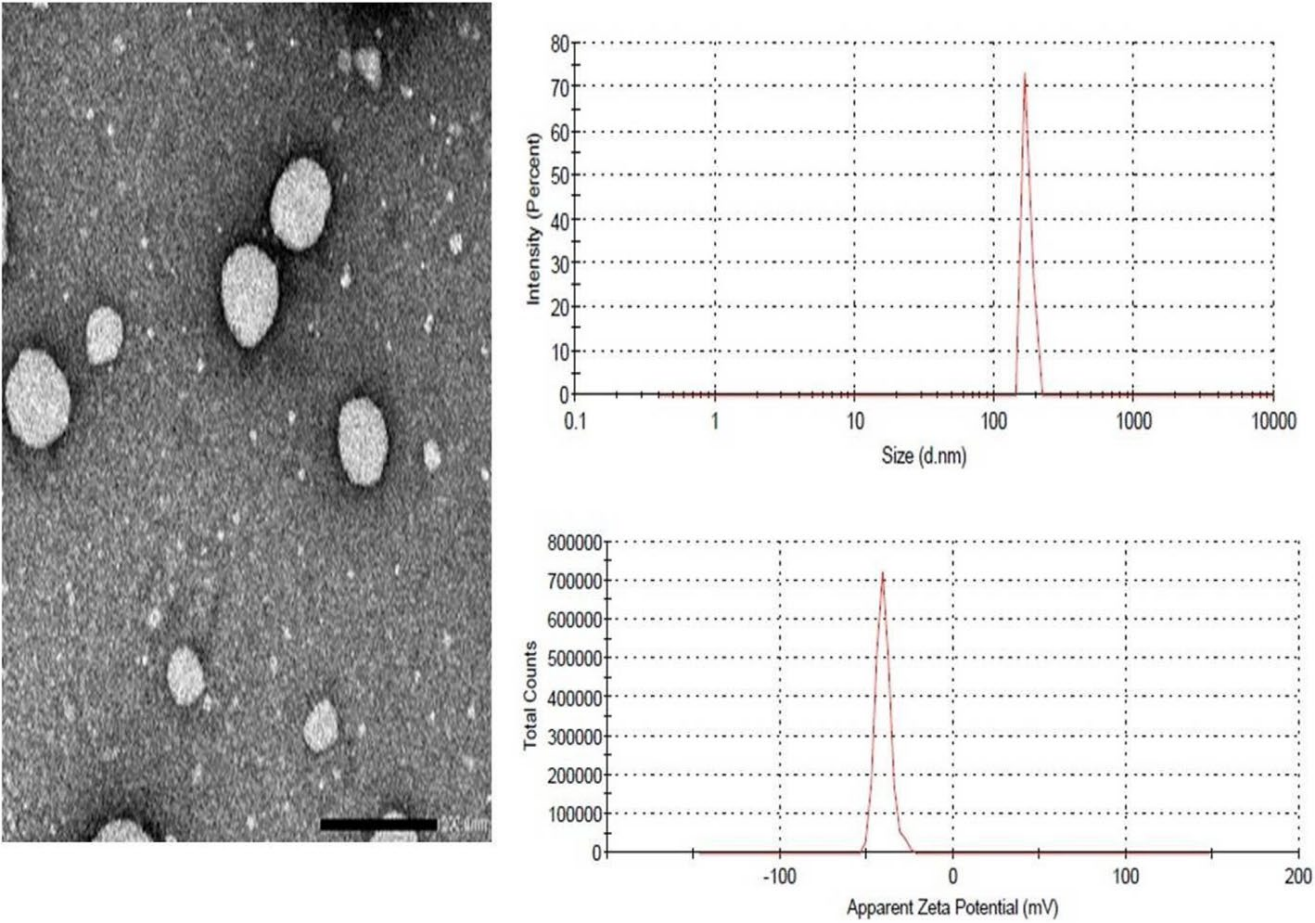
TEM image and droplet size analysis of t-RSV-loaded nanoemulgel (F1)

Droplet size examination was done to prove the formation of nanoemulsion droplets and uniform distribution. All nanoemulgel formulations were nano-sized in the range of 180.3 to 263.5 nm. The polydispersity index (PDI) values were low (0.31–0.48), indicating narrow distribution of droplet size within the formulations. All formulations expressed relatively high ZP ranging from − 40.4 to − 46.9 mV confirming superior stability (Table 2). The TEM image of t-RSV-loaded nanoemulgel (F1) exhibited a regular, completely spherical to subspherical shape in the nanometer size range (Fig. 5).

#### Rheological studies and spreadability test

**Fig. 6:**
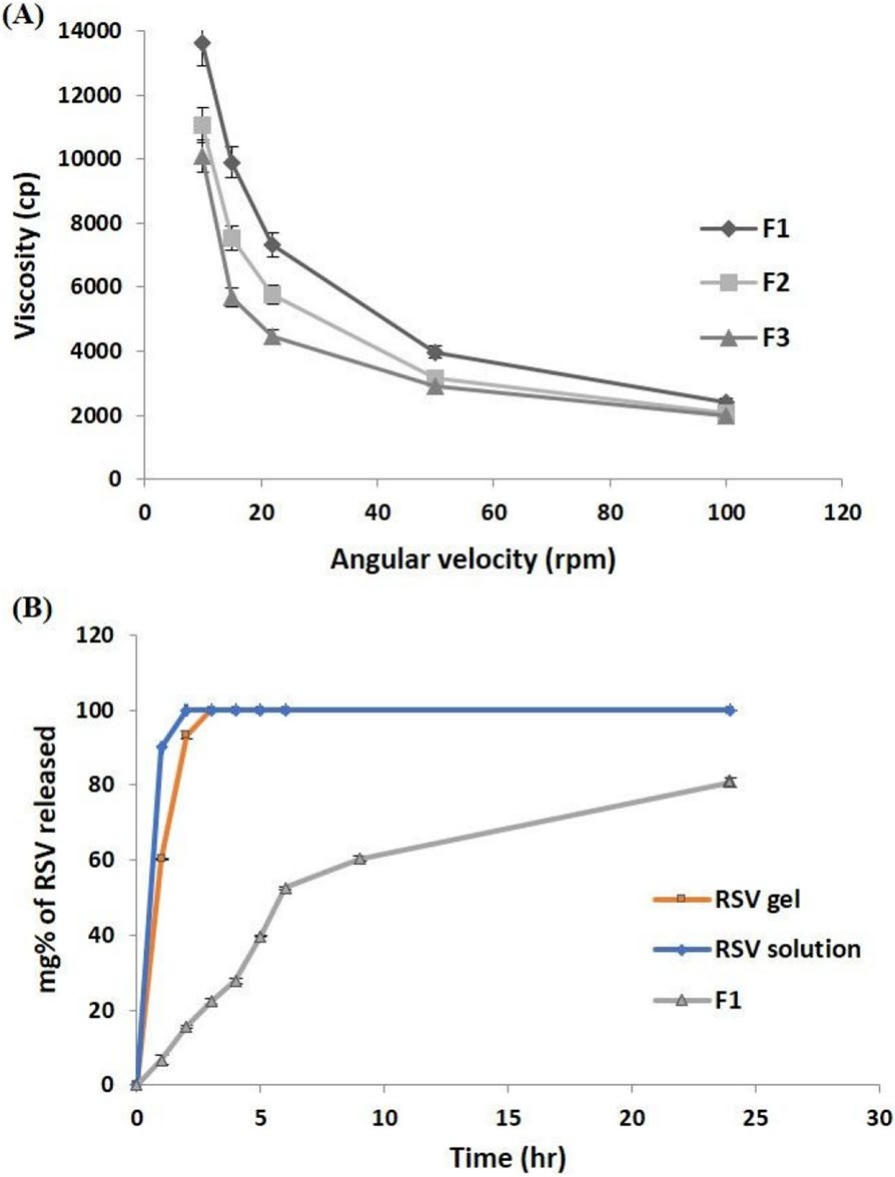
**A** Rheogram of t-RSV-loaded nanoemulgels; F1, F2and F3at room temperature & (**B**) *In-vitro* release behavior of t-RSV-loaded formulations in phosphate buffer, pH 7.4 at 37 °C

Viscosity measurement of nanoemulgels (F1, F2 and F3) indicated pseudo-plastic rheology, as confirmed by shear thinning and a decline in viscosity with increased angular velocity (Fig. 6A). The apparent viscosity at 50 s^−1^ and 25°C was 3980 cp for F1, 3180 cp for F2 and 2890 for F3. Thespreadability results indicated smooth spreading by small amount of shear: F3 (4.5 cm) > F2 (3.8cm) > F1 (3.5 cm). According to the obtained results, the selected formulation for further investigations was F1 which showed the smallest particle size (180.3 nm) with high ZP value; -46.9 mV and desirable viscosity and spreadability.

#### *In-vitro* release study

Drug released from t-RSV-loaded nanoemulgel (F1) was 27.73, 52.57and 80.67% after 4, 6 and 24 h. respectively, indicating sustained release behavior (Fig. 6B), whereas t-RSV solution and t-RSV gel showed a very rapid release of 99.97% and 93.3%; respectively after only 2 h. The *in-vitro* release results were analyzed and fitted kinetic models to explain the release kinetics. The results obtained were best fitted to first-order release kinetics with *R*^*2*^ value (0.965 ± 0.001).

#### Stability testing

**Fig. 7:**
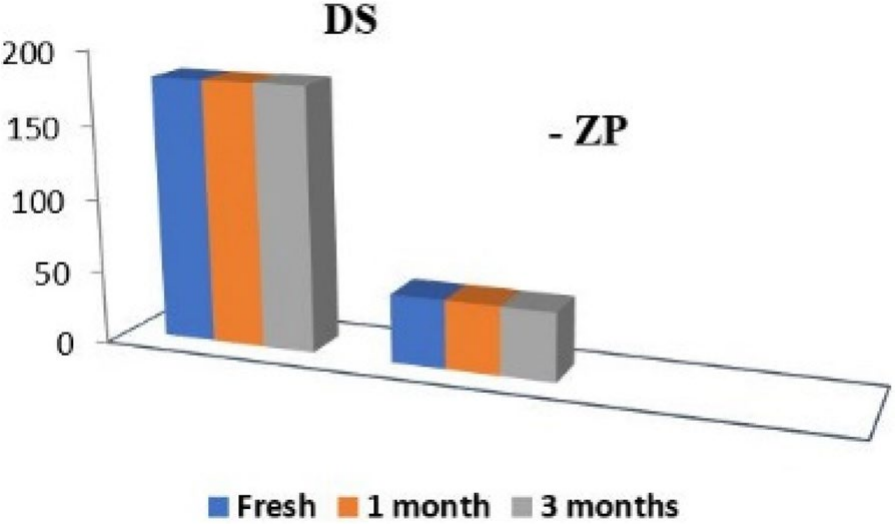
The change in particle size (DS) and zeta potential (ZP) for 3 months

The optimized formulation (F1) showed suitable properties with no phase separation following the high centrifugation speed of 10,000 rpm for5 minutes. It embraced superior stability at 4ºC during 3 months, with reference to the organoleptic characters; appearance, odor and uniformity were revealed. In addition, no significant change in DS, ZP or PDI was observed during the study period (*p* > 0.05, Paired Student T-test) (Fig. 7).

## Discussion

UTIs are among the most prevalent infections worldwide UPEC is responsible for about 80–90% of UTIs cases [40].The current increase of multidrug resistant UPEC strains makes treatment for UTIs a major challenge [41].

Variety of natural compounds have been studied to explore their therapeutic potential for both preventing and treating disease. The naturally occurring phytoalexin, t-RSV, has a wide range of biological effects, including antibacterial, antiviral, antioxidant, anti-inflammatory, anti-aging, anti-carcinogenic, and neuroprotective qualities. Common sources of t-RSV (3,5,40-trihydroxystilbene) include grapes, red wine, peanuts, and a number of woody plants [42, 43].

Pathogenic bacteria often express tightly regulated virulence genes to adapt to their environment timely and efficiently [44]. The rationale for applying anti-virulence molecules to therapy is to disarm the pathogen of its abilities to harm the host, allowing the immune system to eradicate it [12]. Here we confirm that t-RSV at subinhibitory concentrations can effectively lower the expression of virulence factors of UPEC clinical isolates. Several studies have reported that Gram-negative bacteria, such as *E. coli*, have been shown to have lesser sensitivity to t-RSV compared to Gram-positive pathogens with MIC values > 200 µg/mL [45, 46]. This observation could be the result of t-RSV's active extrusion via efflux pump systems or it could be the result of t-RSV's limited penetration of certain Gram-negative bacteria across the outer membrane [47]. In our study, the MIC of t-RSV on the studied *E. coli* isolates was 500 µg/mL, which was comparable to previous values reported for *E. coli* isolates [6, 48, 49]. RSV exhibits antibacterial action against *E. coli* and other bacteria through damage the cell membrane and inhibition of the bacterial energy metabolism. Other possible targets of RSV in *E. coli* are cell-division protein FtsZ and DNA gyrase [16, 48, 49].

As oppose to their commensal status as intestinal flora, UPEC strains grow and persist in the urinary tract and exhibit a range of virulence factors and strategies that enable them to cause infection and disease [50]. Adhesins or fimbriae, biofilm formation, and toxins are the most important of UPEC virulence factors [10]. Our study investigates the phenotypic expression of UPEC virulence factors and the existence of the virulence genes as well. In the late 1970s, it was first discovered that *E. coli* strains that cause UTIs often agglutinate human RBCs despite the presence of mannose, and that this was mostly mediated by fimbriae. Following that, a number of virulence factors were proposed as virulence markers for UPEC isolates [51].

Microbial communities of organisms that are firmly attached to one another or a target surface are known as biofilms [52]. UPEC biofilms provide nutrient-rich conditions for growth and persistence of microorganisms at infection sites, and they protect bacteria from antibiotics phagocytosis, host defense mechanisms, and hydrodynamic flow in the urinary tract [4, 50, 53]. In the current work, 50.9% of the investigated isolates were positive biofilm- producers which was close to a study by [54] that reported biofilm formation of 56.52% in acute UTIs. Other studies showed lower biofilm production than reported in our study [55, 56]. The attachment of UPEC to urinary tracts and the development of biofilms involve several adhesion factors [57]. In our study, 29 isolates were positive for *BssS* gene. It was found that 16 out of the 28 biofilm producing isolates harbor the *BssS* gene with 7 of them containing *papC* gene as well, while 1 isolate harbor the *papC* gene alone. On the other hand, 11 biofilm producing isolates did not harbor either of these genes. The existence of additional virulence genes involved in the production of biofilms may help to explain this [57]. In addition, t-RSV (100 µg/mL) decreased biofilm formation in all positive isolates ranging from (9 – 69%). This was in line with results obtained by Lee et al. [6], which revealed that t-RSV and oxy-RSV at 100 µg/mL inhibited UPEC biofilm formation by > 80%. In addition, another study [58] showed that t-RSV at 100 µg/mL induced a 39.85% suppression of the development of MRSA biofilms. The antibiofilm effect in *E. coli* is caused by decreased expression of the genes (csgA and csgB) that code for fimbrial development, which is necessary for biofilm formation [59, 60].

Hemagglutination, a critical virulence factor in the UPEC infections, is mediated by fimbriae [61]. The most common fimbriae found in UPEC strains are type I (MSHA); responsible for bladder infections, and type P (MRHA); responsible for pyelonephritis. These fimbriae are crucial for adhering to and invading bladder and kidney epithelial cells [1, 4]. The current study revealed that 23.6% of tested isolates showed both MRHA & MSHA haemagglutination (HA) of group O RBCs. This was close to studies of Fatima et al. (30%), Kausar et al. (30%), Raksha et al. (30.9%), and Varshney and Dimri (31.9%) [61–64], while Green & Thomas (56%) and Johnson (58%) [65, 66] reported higher rates of MRHA positivity. For MSHA, our results differ from a previous study [67], which reported 14% MSHA and [62], showing 12.9% MSHA among the studied isolates. In the current study, *FimH* and *PapC* genes were detected in 21 and 14 isolates, respectively with only 5 of the 13 MSHA positive isolates and 8 of the 13 MRHA positive isolates harbor these genes, respectively. This was in line of other studies which reported that type I [6] and type P fimbria [32] can be encoded by other genes. In the present investigation, a concentration of 100 µg/mL of t-RSV markedly inhibited RBCs agglutination in all positive isolates. This coincides with results obtained by Lee et al. [6], who reported that at a concentration of 100 µg/mL of both t-RSV and oxy-RSV, RBCs agglutination by UPEC was markedly inhibited.

Normal serum is typically thought to be an essential component of the host's defense against bacterial infections due to its bactericidal effect. Some *E. coli* strains may be resistant to serum and phagocytes due to the capsule/K antigen on the cell surface [68]. Our results showed that 21.8% of tested isolates were serum resistant. These results were less than those reported in a previous studies [67, 69] that showed a higher percentage of resistance towards serum of *E. coli* isolates (100% and 67% respectively). In our work, 21 isolates were harboring *iss* gene with 9 out of the 12 serum resistant isolates harbor the gene. This could be explained by the fact that other genes, e.g., *traT* gene, can mediate serum resistance in UPEC [70]. Regarding the effect of t-RSV on serum resistance, it was revealed that t-RSV could markedly decrease the serum resistance of serum resistant isolates ranging from (50–95%). The lytic activity of the alternative complement pathway allowed normal human serum to kill bacteria [71]. Bacterial resistance to killing by serum, results from the individual or combined effects of the capsular polysaccharide, O polysaccharide, and surface proteins. Serum resistance may possibly be related to the sialic acid content, which decreased the bacterial surface's capacity to activate and complement by an alternative pathway [72]. We can conclude that t-RSV decreased serum resistance of the tested isolates by decreasing sialic acid content and this could activate the alternative complement system.

Moreover, UPEC strains often produce exotoxins such as hemolysin, CNF-1, and colonization factors [73]. In particular, UPEC strains with higher virulence produce hemolysin [4, 50] Alpha-hemolysin is the cytolytic protein toxin released by the majority of hemolytic *E. coli* strains. *E. coli* also produced cell-associated lysin on blood agar plates and hemolysin caused a clear zone of lysis [74]. In our study, hemolysin production was observed in 18.2% of isolates, which is less than findings of Johnson (38%) and Varshney and Dimri (33.34%) [62, 66]. Also, Raksha et al. (41.36%) and Brook et al. (43%) [61, 75] found slightly higher incidence of hemolytic strains in their study. Moreover,[76] found a much higher percentage (76%) of hemolytic UPEC strains in their study. The lower incidence of hemolysin and serum resistance in our study could be explained by the fact that most of the strains were isolated from cases of cystitis rather than pyelonephritis. In the current study*, hly-A* gene was detected in 10 isolates with all the 10 hemolysin producing isolates detected phenotypically were harboring the gene. It was found that t-RSV in a concentration of 100 µg/mL decreased hemolysin production in all positive isolates from 13.2 – 96.8%. In other studies, [77, 78], hemolytic activity of *Staphylococcus auerus* isolates was markedly reduced when treated with t-RSV.

Regarding cytotoxic effects of *E. coli* in this investigation, it was revealed that 36.4% of the isolates showed cytotoxic effect on HEp-2 cell line with varying degree. Rounding of the HEp-2 cells was observed as early as 1 h after infection in all positive samples. while complete detachment of the cells was observed 3 h after infection. These results were less than obtained in other studies [33] which reported that 92% of isolates showed cytotoxic effect. MTT assay was used to confirm the cytotoxic effect of *E. coli* isolates on HEp-2 cell line. Results revealed that 20 isolates have cytotoxic effect on HEp-2 cell after 2h of infection ranged from 12–35%. It was found that 11 isolates harbor *cnf-1* gene with all of them showed cytotoxicity on HEp-2 cells, however, 9 isolates showed cytotoxicity but did not harbor the gene. This was reported in previous studies where other UPEC proteins were shown to elicit toxic effects on host cells [3]. Additionally, results showed that t-RSV decreased the cytotoxicity of positive isolates in a range from 9- 77%.

In our work, it was found that there is a statistically significant association between the presence of both *papC* and *cnf-1* and the presence of *hly-A* and *iss* genes (*p* value ≤ 0.05). This was in line with previous studies which show that hly*-A* gene is present in almost all UPEC strains to produce *cnf-1*. This can be explained by that the genes encoding cnf-1, α–hemolysin and P fimbriae are found on the same pathogenicity island in some strains of UPEC [29]. It is suggested that *cnf-1* and hemolysin are involved in virulence mechanisms that benefit the bacteria [73]. In addition, there is a statistically significant association between the presence of *BssS* gene and both *FimH* and *iss* genes (*p*-value ≤ 0.05). Moreover, the current work showed that pyelonephritis is statistically associated with *hly-A*, *cnf-1* and *papC* genes (*p*-value = 002, 0.01 and 0.03, respectively).

Additionally, we investigated how t-RSV affected the gene expression of several UPEC virulence genes including *BssS, fimH*, *papC*, *iss, hly-A* and *cnf-1*. Results showed that t-RSV significantly downregulates the gene expression of all tested genes. Our results coincide with a previous study [6] which reported that t-RSV and oxy-RSV at 50 µg /mL significantly decreased the expression of *csgA*, *csgB*, *FimA*, *FimH*, *motA*, and *papA* genes,. In addition, another study reported that t-RSV repressed curli genes and motility genes in enterohemorrhagic *E. coli* O157:H7 [79].

In the current study, t-RSV-loaded nanoemulgel formulation was prepared and characterized. The optimum selection of the utilized surfactant in emulsion preparation has a significant role in its stability [80]. The safety data of surfactants and co-surfactants is a critical factor in designing emulgels [81]. Surfactants utilized in this research were Tween 20 and Tween 80 which are considered as pharmaceutically accepted and known as generally regarded as safe ‘GRAS’. In addition, the hydrophilic–lipophilic balance (HLB) value of the chosen surfactants was in the range of 14–15, which fulfills the necessity of minimum HLB value for the formulation of a stable o/w nanoemulsion [82]. Propylene glycol was added in the preparations as a co-surfactant and humectants.

Nanomulgel merges the advantages of both gel and emulsion and performs as a reservoir of drugs as emulsion droplets permit inclusion of lipophilic drugs [19]. Nanoemulgel is prepared by establishing nanoemulsion included into a hydrogel matrix [83]. In the current work, t-RSV was fabricated as oil-in-water (o/w) nanoemulsion in a gel phase with droplet size ranges from 180.3 to 263.5 nm. The selected formulation (F1) showed the smallest DS of 180.3 nm. It could be observed that the DS measurement obtained by TEM for F1 is smaller than that found by light scattering process. This result agreed with various researchers [84–86] who correlated the reason to the method of sample preparation for TEM evaluation which contains dehydration of the nano-formulation. Whereas zetasizer determines the apparent size which includes the aqueous layers nearby the nanoparticles. The PDI reflects droplets size distribution uniformity and it ranges between 0 and 1. The closer its value to zero, the greater is the similarity between the particles. F1 exhibited the smallest droplet size, the lowest PDI value and the highest zeta potential of 180.3, 0.31 and 46.9 mV, respectively. The zeta potential is investigative to the nanoformulations’ stability versus agglomeration. The higher the absolute value of zeta potential, the more stable the nanoformulation system. Nanoemulsions have a zeta potential between + 100 to -100 mV. Neutral nanoemulsions exhibit zeta potential value ranging from -10 to + 10 mV [87]. The high colloidal stability of t-RSV- loaded formulations is due to the electrostatic repulsion between approaching oil droplets which can hinder coalescence [37].

The viscosity affects spreadability, syringeability and the appearance of the formulations. The shear thinning behavior observed for t-RSV-loaded nanoemulgels is a favorable property since it should exhibit smooth flow for the period of application intra-urethral by the needle-less syringe [35]. This pseudoplasticity could be a result from the colloidal network arrangement which aligns itself in the direction of shear, thus reducing the viscosity as the shear rate elevates [88]. t-RSV-loaded nanoemulgel (F1) showed viscosity of 3980 cp with acceptable spreadability value. The high viscosity of t-RSV-loaded nanoemulgel can enhance the contact time of the formulation at the site of action [89].This finding suggested the significance of our formulation (F1) for future *in-vivo* studies enhancing the sustained effect of t-RSV.

The selected t-RSV-loaded nanoemulgel (F1) exhibited significant (*p* < 0.05) delay in the initial drug release compared to t-RSV gel and t-RSV solution (Fig. 6). t-RSV-loaded nanoemulgel (F1) released only about 15% of the loaded t-RSV after 2 h. The observed high drug release from both t-RSV gel and solution might be due to the unstable liquid consistency which leads to promoted drug leakage. Alternatively, the slow-release rate of t-RSV from (F1) might be attributed to the fact that the drug-loaded inside the oil droplets which are surrounded by the polymer coat, and thus the drug had to transfer through a long and tortuous pattern to achieve the release medium. Comparative results were obtained by Ferreira S. et al. [90] for curcumin, a poorly water soluble drug from the prepared nanoemulgel formulation, where only 30% of drug was released after 8h [19]. Furthermore, the slow release rate of t-RSV from the nanoemulgel could be attributed to the small aqueous content of the solvent mixtures and the inclusion of liquid paraffin [91].The *in-vitro* release data for F1 best fitted to first-order kinetics, where the release rate depends on the drug concentration. Comparable results were obtained with Yeo, E. et al. [92], where all nanoemulgel formulations fitted first order kinetics.

Figure 7 shows that there is no significant deviation in DS or ZP after 3 months for F1. This stability could be related to the homogenization factors which were sufficient to disperse the oily phase as fine droplets [35]. In addition, the developed surface charge presented by the high ZP resulted in a valuable repulsive electrical forces among impending oil droplets hindering the coalescence [36]. Moreover, the high viscosity of the gel consisting the external phase greatly constrained the Brownian action [93].

## Conclusion

Our study proves the *in-vitro* antivirulence effect of subinhibitory concentrations of t-RSV on clinical UPEC strains and successfully developed and characterized a stable t-RSV-nanoemulgel. To our knowledge, this is the first study to assess the antivirulence activity of -t-RSV on a large number of clinical UPEC isolates. These results together with the results obtained in previous studies place this natural compound to be a potential good alternative in the treatment of persistent UTIs where antibiotic resistance is imposing a serious problem. However, these results have to be proved *in-vivo* through animal studies. The newly characterized t-RSV formula in this study shall encourage the trial of this compound in an animal model with persistent UTI through applying the formula via intrauretheral route. Limitations of the study include the lack of testing of the newly developed nanoemulgel on UPEC strains.

## Data Availability

The original contributions presented in the study are included in the article, further inquiries can be directed to the corresponding author.
